# To study the utility of COX-2 as immunohistochemical prognostic marker in comparison to various histopathological parameters and TNM staging in breast carcinoma: an observational, cross-sectional study protocol

**DOI:** 10.12688/f1000research.138776.2

**Published:** 2024-06-18

**Authors:** Jayashree Bhawani, Samarth Shukla, Sourya Acharya, Sunita Vagha, Miheer Jagtap

**Affiliations:** 1 Department of Pathology, Jawaharlal Nehru Medical college, Sawangi(Meghe), Wardha, Maharashtra, 442001, India; 2 Department of General Medicine, Jawaharlal Nehru Medical College, Sawangi(Meghe), Wardha, Maharashtra, 442001, India

**Keywords:** Breast Cancer, COX-2, histopathological parameters, prognostic marker, immunohistochemistry.

## Abstract

**Background:**

Breast cancer is the most prevalent cancer among women worldwide and is a well-known cause for cancer mortality in females. COX-2 (cyclooxygenase) plays a vital role in development of some human cancers such as lung, colon and breast. It is a potent enzyme that is important for the conversion of arachidonic acid into prostaglandins. These prostaglandins mediate cellular proliferation, apoptosis and angiogenesis which contributes to carcinogenesis. Overexpression of COX-2 has been detected in several malignancies including breast cancer. COX-2 overexpression is regarded as a poor prognostic marker of breast cancer.

The present study will aim to study the immunohistochemical expression of COX-2 in breast cancer and compare it with known histopathological parameters thus assessing its prognostic value.

**Methods:**

This will be an observational study conducted in the Department of Pathology, JNMC, Wardha (Sawangi). Radical mastectomy specimens will be studied for COX-2 expression by immunohistochemistry in patients diagnosed with breast carcinoma. COX-2 expression will be quantified as immunohistochemical score and results will be correlated with various histopathological parameters.

**Results:**

The expected result of our study will suggest an association of COX-2 expression to the factors associated with poor prognosis in breast carcinoma. A positive correlation is expected between larger tumor size, positive lymph node status, higher T stage and N stage and lymphovascular invasion.

**Conclusions:**

Conclusions will be drawn from the obtained results of the immunohistochemical study by using COX-2- for detection of overexpression of COX-2 when evaluated with TNM staging, histological grading and molecular types of breast cancer.

## Introduction

Breast carcinoma is the most frequent malignancy and the second leading cause of death in women worldwide. ^1^ It has surpassed lung cancer with an estimated 2.3 million new cases worldwide. In recent years, incidence rates of breast cancer have elevated by 0.5% every year, as breast cancer incidence varies with age and race/ethnicity.

In the year 2021, 2.6 million women were newly diagnosed with breast cancer. ^2^ In India, breast cancer accounts for 13.5% of cancer cases. It is assumed that the chance of a woman’s death due to breast carcinoma is about 1 in 39 or about 2.6%. ^3^

The etiology is multifactorial, implicating hormonal imbalance, genetic predisposition, diet, metabolic factors, and reproductive factors.

The diagnosis of breast cancer is achieved by adopting a triple assessment approach which includes clinical evaluation, radiographical investigation and tissue diagnosis by biopsy, out of which tissue biopsy is currently the gold standard for cancer diagnosis and predicting prognosis.

There are certain indicators available which establish the prognosis of breast cancer, ^4^ these are tumor size, age, site of tumor, lymph node status, stage and grade of tumor and lymphovascular invasion.

Various evaluation methods or systems are available for the determination prognosis of breast carcinoma, including the: - Nottingham Histologic Grading system which is also referred as The Elston-Ellis modification of Scarff-Bloom-Richardson grading system. The other method is the categorization of breast cancer according to TNM (tumor, nodal status, metastasis) staging, specified by the American Joint Committee on Cancer (AJCC). There is a more advanced diagnostic modality, which determines the molecular phenotypes of carcinoma breast by implementing immunohistochemistry.

Over the years additional prognostic and predictive factors are evolving and one of them is COX-2. This is the rate limiting enzyme in the biosynthesis of prostaglandins from arachidonic acid. ^5^ It is an empirical catalyst formed under few definitive circumstances of inflammation and tumor -microenvironment. COX-2 is an important tumor marker which regulates tumor growth, invasion and metastasis. ^6^ In carcinogenesis, inflammatory cytokines, growth factors and oncogenes trigger induction of COX-2, which results in tumor progression. COX-2 is strongly associated with tumor proliferation, angiogenesis and apoptosis resistance mechanism. ^7^ Therefore, COX-2 overexpression is associated with poor prognosis in post-menopausal women, those above 50 years of age, those in the advanced stage of the disease, those with a larger tumor size, higher grade, and metastasis of lymph nodes.

Various studies ^8^ showed that, among different types of breast tumors, triple negative breast cancer [ER (estrogren) negative, PR (progesterone) negative, HER2 neu negative)] are associated with aggressive progression of tumor and COX-2 expression seemed to be increased in triple negative tumors. Also, there is a positive correlation of COX-2 with these histopathological prognostic parameters.

To further evaluate the role of COX-2 as marker of tumor behavior and to correlate its expression with other essential parameters which play a vital role in the assessment of tumor behavior this study is being conducted.

The aim of this study is to analyze the relationship between COX-2 expression and histopathological parameters, pathological TNM staging and molecular immunophenotypes of carcinoma breast.

### Objectives

• To confirm breast carcinoma by histopathological examination and to determine the histopathological grade of breast carcinoma by Elston-Ellis modification of Scarff- Bloom-Richardson grading system).
• To establish pathological TNM stage of breast carcinoma.
• To determine the immunophenotypes of carcinoma breast by evaluating ER/PR and Her 2 neu expression by immunohistochemistry.
• To evaluate immunohistochemical expression of COX-2 in breast carcinoma.
• To understand the inter relation between expression of COX-2, histopathological prognostic markers, molecular immunophenotypes, and pathological TNM staging of carcinoma breast.

## Methods

### Study design

An observational, cross-sectional study will be conducted on breast cancer patients for a span of two years (June 2022 to June 2024) in the Histopathology and Immunohistochemistry section of the Pathology Department, Jawaharlal Nehru Medical College, Sawangi (Meghe) in co-ordination with General Surgery Department, Acharya Vinoba Bhave Rural Hospital, Sawangi (Meghe).

### Sample size

Sample size calculation according to formula:

$$n=Z{\left(1-\alpha /2\right)}^{2}\times p\times \left(1-p\right)/d^{2}$$

here,

Z (1 − *α*/2) = 1.96 (value of the standard normal variate corresponding to the level of significance *α* of 0.05)

p = Expected prevalence of COX-2 expression in the study population (76% or 0.76) ^9^

d = Specified precision on either side of the mean (10% or 0.1)

$$\begin{array}{c} n=\left(1.96\times 1.96\right)\times 0.76\times \left(1-0.76\right)/\left(0.1\times 0.1\right)=70.07\\ =\text{Approximately 70–80 patients needed in each study group}. \end{array}$$

The study will include approximately 70-80 resected specimens from confirmed and planned modified radical mastectomy of breast carcinoma in the General Pathology Department, J.N.M.C.

### Ethics and consent

The Institutional Ethics Committee has given approval for the study ((DMIMS (DU)/IEC/2022/1068) on 27 ^th^ June 2022), and informed consent will be taken from the targeted patients who will be participating in this study.

### Inclusion criteria

• Female patients diagnosed with breast carcinoma on histopathological examination.
• Women with breast carcinoma having no history of treatment.
• Female patients presenting with breast carcinoma arising *de novo.*
• All cases that underwent mastectomy/modified radical mastectomy (MRM) procedure.

### Exclusion criteria

• Patients with lesions other than breast carcinoma i.e., benign breast tumors, myoepithelial tumors, mesenchymal tumors.
• Patients with breast carcinoma who have received neoadjuvant chemotherapy.
• Male patients with a diagnosis of breast carcinoma.
• Patients who underwent incisional biopsy, excisional biopsy, or tru-cut biopsy.

### Approach to the study

The study will collect patient’s personal details including name, age, gender, registration number, IPD/OPD numbers, department and also clinical details such as symptoms, test interpretations and diagnosis. Radiological investigations such as mammography report, ultrasonography of breast, and CT scan reports will be noted from patient’s case file. The present study will take into consideration, the biopsy report of the lesion.

The mastectomy specimen will be received in the Histopathology Department, in 10% formalin and will be kept for proper fixation. After a period of 24 hours of fixation the specimen will be grossed and sections from the representative areas will be taken.

### Method of tissue processing ^10^

The sections will be processed by automated histokinette. The paraffin blocks will be prepared with the help of Leuckhart’s mould and sections of 3-4 microns thick will be taken using microtome. These sections will be then transferred to glass slides, precoated with egg albumin. Hematoxylin and eosin staining of paraffin sections will be carried out using standard methods. Slides will be scanned and a diagnosis along with histological grading will be made, using the College of American Pathologists (CAP) protocol. The 8 ^th^ American Joint Committee on Cancer (AJCC) guidelines will be followed to carry out staging on the specimen.

### Methodology of interpretation

Using the Nottingham modification of Bloom-Richardson System, histological grades of tumor will be determined. This will include factors such as tubule formation, nuclear size and mitotic count ^11^ and score will be assigned accordingly:

Tubule formation

Score 1: > 75% of tumor shows tubules

Score 2: 10-75% of tumor has tubules

Score 3: < 10% of tumor has tubules

Nuclear size

Score 1: Small regular nuclei: similar to normal ductal nuclei (nuclei with minimal variation in size and shape)

Score 2: Intermediate size: 1.5-2 times the size of normal ductal nuclei (nuclei with moderate variation in size and shape)

Score 3: High grade nuclei: > twice the size of normal ductal nuclei (nuclei with marked variation in size and shape)

Number of mitotic figures counting 10 high power fields ( Table 1):

**Table 1.** Score Categories According to Field Diameter and Mitotic Count. ^12^

Scoring categories of mitotic counts				
Final diameter (mm)	Area (mm ^2)^	Number of mitosis per 10 fields corresponding to:		
		Score 1	Score 2	Score 3
0.44	0.152	0-5	6-10	>11
0.59	0.274	0-9	10-19	>20
0.63	0.312	0-11	12-22	>23

Reproduced from Dooijeweert *et al*. (2022) under a CC-BY-4 license.

**Table 2.** Evaluation of COX-2 immunostaining status based on proportion score i.e percentage of positive cells. ^1^

Percentage of positive cancer cells	COX-2 quantity score
No staining	0
1%-10%	1
11%-50%	2
51%- 80%	3
>81%	4

Reproduced from Solanki *et al*. (2018) under a Creative Commons Attribution-NonCommercial 4.0 International License.

**Table 3.** Evaluation of COX-2 immunostaining status by measuring intensity of staining. ^1^

Staining intensity	Staining intensity score
No staining	0
Weak staining	1
Moderate staining	2
Strong staining	3

Reproduced from Solanki *et al*. (2018) under a Creative Commons Attribution-NonCommercial 4.0 International License.

Score 1: 0-9 mitoses/10 HPF

Score 2: 10-19 mitoses/10 HPF

Score 3: > 20 mitoses/10 HPF

Add up to the score to reach the total points:

1-5 points: grade 1: well differentiated

6-7 points: grade 2: moderately differentiated

8-9 points: grade 3: poorly differentiated

After assessing tumor grade, tumor stage will be determined by TNM staging (8 ^th^ AJCC edition)

Hence, interpretation of overall stage will be as follows:

**Stage 0**: Tis

**Stage I**: T1, N0

**Stage II**: T2, N0; T3, N0; T0, N1; T1, N1 or T2, N1

**Stage III**: T3N1, T0N2, T1N2, T2N2, T3N2, any T-N3, locally advanced carcinoma breast, invasion in ribs, skin, matted lymph nodes

**Stage IV**: M1; advanced breast cancer.

### Immunohistochemical assessment ^13^

Immunohistochemistry (IHC) will be performed on a paraffin embedded segment of a surgically removed specimen that has been diagnosed as a malignant lesion of the breast. Only representative blocks from the lesion will be chosen for immunohistochemistry. Immunohistochemistry will be performed utilizing monoclonal antibodies and the biotin avidin peroxidase complex technique. For performing IHC, slides will be coated with Poly L- Lysine and 2 sections of 3-4 microns thick will be placed on the slide. Sections will be deparaffinized and then rehydrated with descending grades of alcohol (100% to 90% to 70% to at last 50% concentration). Antigen retrieval will be performed and followed by transfer of slide for wash in tris buffer solution (TBS). The next step will be the peroxidase blocking for 30 minutes using 3,5 hydrogen peroxide along with methanol. The wash will be repeated 3 times with TBS. Now the section will be exposed to DAKO monoclonal mouse anti human antibodies, i.e., mouse anti human ER/PR/Her2 antibodies and COX-2 antibody. It will be again washed in the buffer thrice for 5 minutes. Now chromogen 3,3’- diaminobenzydene (DAB) will be applied for 15-20 minutes. The DAB-stained slide will be washed in buffer for 10 minutes and then will be dipped in distilled water. Harris hematoxylin will be used for counterstaining. Lastly, after final wash with tap water, mounting of slides will be done to scan under microscope. ER/PR/Her 2 scoring will be assessed according to Allred scoring method mentioned in CAP guidelines. ^14^

### Evaluation of COX-2 expression by immuno-histochemistry

On immunohistochemical staining COX-2 will be observed in tumor cells. Immunohistochemical staining will be evaluated with a light microscope. A minimum of 500 cells will be counted per x 40 fields for immunohistochemical evaluation per antibody. Cyclooxygenase-2 positivity will be indicated by the presence of brown cytoplasmic- staining. For the evaluation of cytoplasmic staining for COX-2, a predefined scoring system which is based on the product of percentage of immunoreactive tumor cells with staining intensity will be studied. ^1^ Immuno-reactivity for C0X-2 in tumor cells will be assessed using a scoring system based on the staining intensity.

COX-2 immunohistochemistry score (HIS) is obtained by multiplying the staining quantity score i.e. values according to Table 2 and staining intensity score i.e. values obtained according to Table 3. Obtained value after multiplication will be put under 3 categories which are:

0-3 = Negative/faint staining

4-8 = Moderate/intermediate staining

9-2 = High staining

### Statistical-analysis

The software used for analysis will be SPSS version 27.0. Statistical analysis will be carried out by using suitable statistical tests eg: ‘Chi-Square Test’, ‘Fisher’s Exact Test’ with significance set at p value < 0.05.

## Results

Observations and results will be collected and combined together over the period of two years and will be analyzed statistically.

## Discussion

In this study a statistically significant expression of COX-2 with histopathological parameters which are associated with poor prognosis such as tumor size, grade, lymph node metastasis is expected. As COX-2 is overexpressed in triple negative breast cancer, it indicates a poor outcome in terms of prognosis. COX-2 expression is increased when tested in post- menopausal status, those of age above 50 years, in an advanced stage of disease, with a larger tumor size, a high grade, and metastasis of lymph nodes *.* Few studies have shown that COX-2 expression is increased when correlated with HER-2/neu expression in breast cancer patients; COX-2 was expressed in ER negative, PR negative and HER-2/neu positive tumors and with high histological grade in breast carcinoma. Our study will help in assessing the role of COX-2 in carcinogenesis and its association with poor prognostic markers.

A key strength of this study is that it will use a useful biomarker for estimating tumor aggressiveness. This study will focus on COX-2 expression in breast carcinoma and its comparison with other histopathological parameters. This would also give clinicians a framework for evaluating the function of selective COX-2 inhibitors in human breast cancer chemoprevention as well as treatment.

Studies such as those conducted by Ibrahim Metin Ciris *et al.*, Debashri Jana *et al.*, Ari Ristimaki *et al*., Pereira *et al*. and Costa *et al*. have stated that COX- 2 overexpression has the potential to be a useful biomarker for estimating tumor aggressiveness, and that COX-2 expression is associated with aggressive tumor biology, and can behave as a predictor of tumors with bad prognosis. Hence, the above studies concluded that there is a correlation of COX-2 expression with histopathological prognostic parameters in breast cancer. The limitation of the study is that it will be a single center study.

## Conclusion

The purpose of the current investigation is to determine COX-2's precise prognostic function in breast cancer. This would give clinicians a framework for evaluating the function of selective COX-2 inhibitors in human breast cancer chemoprevention as well as treatment. This study will correlate prognostic relation between COX-2 expression in breast carcinoma patients with routinely studies histopathological prognostic parameters.

### Dissemination

The results of this study will be presented in peer reviewed publications.

### Study status

Recruitment has begun, and currently 25 patients have been enrolled for the study.

## Ethics and consent

The research work is approved by Institutional Ethical Committee of the Datta Meghe Institute of Medical Sciences (DMIMS (DU)/IEC/2022/1068) on 27 ^th^ June 2022.

## Author contributions

Contributors: Dr. Samarth Shukla conceptualized the study and provided the idea of how to conduct it. Dr. Jayashree Bhawani will do the research work, collect the data, analyze it and under guidance of Dr. Samarth Shukla and Dr. Sunita Vagha will come to the conclusion.

## Data Availability

No data are associated with this article.
