# Supplementary material for: To study the utility of COX-2 as immunohistochemical prognostic marker in comparison to various histopathological parameters and TNM staging in breast carcinoma: an observational, cross-sectional study protocol
Source: F1000Res. 2024 Jun 18;12:1057. Originally published 2023 Aug 30. [Version 2] doi: 10.12688/f1000research.138776.2 (PMC11263903; doi:10.12688/f1000research.138776.2)
Supplement: Peer review history

# Reviewer report: Reviewer response for version 2

**DOI:** 10.5256/f1000research.167792.r292485

**Published:** 2024-07-22

**Authors:** Amit Kumar Chowhan

**Affiliations:** 1 Department of Pathology, AIIMS, RAIPUR, RAIPUR, TirupatiCHHATTISGARH, India

No further comments.

Is the study design appropriate for the research question?

Yes

Is the rationale for, and objectives of, the study clearly described?

Yes

Are sufficient details of the methods provided to allow replication by others?

Partly

Are the datasets clearly presented in a useable and accessible format?

Partly

Reviewer Expertise:

Oncopathology

I confirm that I have read this submission and believe that I have an appropriate level of expertise to confirm that it is of an acceptable scientific standard.

# Reviewer report: Reviewer response for version 1

**DOI:** 10.5256/f1000research.152003.r202391

**Published:** 2024-06-05

**Authors:** Shaan Khetrapal

**Affiliations:** 1 Department of Pathology, Hamdard Institute of Medical Sciences and Research, New Delhi, Delhi, India

In exclusion criteria the point of male patients can be omitted as the inclusion criteria specifically states female patients.

 Comparative elaboration of discussion with respect to other studies can be done, which can be beneficial for further prognostic factors.

 Rest well written article for study, to be approved and considered.

Is the study design appropriate for the research question?

Yes

Is the rationale for, and objectives of, the study clearly described?

Yes

Are sufficient details of the methods provided to allow replication by others?

Yes

Are the datasets clearly presented in a useable and accessible format?

Yes

Reviewer Expertise:

Histopathology

I confirm that I have read this submission and believe that I have an appropriate level of expertise to confirm that it is of an acceptable scientific standard.

# Reviewer report: Reviewer response for version 1

**DOI:** 10.5256/f1000research.152003.r202390

**Published:** 2023-10-02

**Authors:** Amit Kumar Chowhan

**Affiliations:** 1 Department of Pathology, AIIMS, RAIPUR, RAIPUR, TirupatiCHHATTISGARH, India

Comment

1. In Introduction it is mentioned ‘This is a more advanced diagnostic modality, which determines the molecular phenotypes of carcinoma breast by implementing immunohisto[1]chemistry’. – Kindly check whether this line has been kept at appropriate place.
2. Sample size should be calculated in reference to prevalence of positivity for COX2 in breast cancer (data to be taken from other similar study). For example, in reference no 5, the positivity of COX2 is mentioned as 42.7% in tumour cells, so while calculating sample size ‘P’ should be 42.7%.
3. First point of Inclusion criteria is • ‘Female patients diagnosed with breast carcinoma on histopathological examination.’
  Therefore, there is no need of keeping an exclusion criteria as ‘Male patients with a diagnosis of breast carcinoma.’
4. Second point of Inclusion criteria is ‘Women with breast carcinoma having no history of treatment.’Therefore, there is no need of keeping an exclusion criteria as ‘Patients with breast carcinoma who have received neoadjuvant chemotherapy.’
5. Third point of Inclusion criteria is ‘Female patients presenting with breast carcinoma arising de novo.’’ Kindly explain what is the exact meaning of ‘’de novo.’’
6. Fourth point of Inclusion criteria is ‘’All cases that underwent mastectomy/modified radical mastectomy (MRM) procedure’’, therefore there is no need of keeping an exclusion criteria as ‘’Patients who underwent incisional biopsy, excisional biopsy, or tru-cut biopsy’’.
7. Under the heading ‘’Evaluation of COX-2 expression by immuno-histochemistry’’, the reference article mentioned is reference no 14, whereas in table 2 and table 3, the reference mentioned is reference no 1, kindly explain the disparity.
8. Under the heading ‘’Evaluation of COX-2 expression by immuno-histochemistry’’, it has been further mentioned as ‘’COX-2 immunohistochemistry score is obtained by multiplying the staining quantity score and staining intensity score. 0-3 =Negative/faint staining 4-8= Moderate/intermediate staining’’, which is not matching with table 2 and table 3, where the scores are 0-4 and 0-3. Kindly explain the disparity.
9. Under the section ‘’Immunohistochemical assessment’’, it has been mentioned that ER/PR/Her2 antibodies and COX-2 antibody will be applied, kindly mention the scoring system for ER/PR/Her2 antibodies also, which will be followed in the study, along with reference.

Is the study design appropriate for the research question?

Yes

Is the rationale for, and objectives of, the study clearly described?

Yes

Are sufficient details of the methods provided to allow replication by others?

Partly

Are the datasets clearly presented in a useable and accessible format?

Partly

Reviewer Expertise:

Oncopathology

I confirm that I have read this submission and believe that I have an appropriate level of expertise to confirm that it is of an acceptable scientific standard, however I have significant reservations, as outlined above.

# Response

**Published:** 2024-06-10

**Authors:** Jayashree Bhawani

**Affiliations:** Pathology, Jawaharlal Nehru medical college, Sawangi (Meghe), Sawangi, Wardha, Maharashtra, India

1. Instead of this is a more advanced diagnostic modality, It should be there is a more advanced diagnostic  modality which is immunohistochemistry that determines molecular phenotype of breast carcinoma.

 2. **Sample Size**

 Using the formula:

**N= https://f1000research.s3.amazonaws.com/linked/653957.image.png**

                                   

 Where:

- **N** = Sample size
- **Z(1-α/2)** = 1.96 (value of the standard normal variate corresponding to the level of significance α of 0.05)
- **d** = Specified precision on either side of the mean (10% or 0.1)
- **P** = Expected prevalence of COX2 expression in the study population (76% or 0.76)
- From the previous study by Fatma Z Abd Elrahman et al., the prevalence of COX2 expression in carcinoma breast patients was found to be 76%. Taking d=10%, the total sample size calculated is 70.07.

                            N= (1.96) ^2^ X 0.76 X (1−0.76)​

                                                           (0.1) ^2^

                                                   

                                             N= 70.07

  Rounding up to the nearest whole number, the required sample size is **70**.

 Reference- Abdel-rahman FZ, Zaky AH, Zedan A, Elsaba TM, Gabr A. COX-2 “Cyclooxygenase 2“as a Prognostic marker in Breast Cancer. SVU-International Journal of Medical Sciences. 2021 Feb 2;0(0):0–0.

 3. Male patients are kept in exclusion criteria because exclusion criteria comes as an opposite if inclusion criteria and also because in males immunophenotyping cannot be performed.

 4. Again here exclusion criteria is considered as exact opposite of inclusion criteria therefore patients with neoadjuvant therapy  are excluded.

 5. Here recurrence cases and secondaries from other malignancy are not counted therefore primary breast malignancy are referred as de novo.

 6. Here I wanted to be specific for considering only mastectomy patient in inclusion criteria therefore opposites are mentioned in exclusion 

 7. I agree with you Sir, reference no. 1 should have been there at the place of 14th

 8.  Here COX-2 immunohistochemical score will be calculated by multiplying the values of Table 2 and Table 3 and values obtained will be put under 3 categories i.e

 0-3 = Negative / Faint 

 4-8= Moderate

 9-12 = High 

 9. ER/PR/Her2 antibodies assessment according to Allred scoring system as depicted in CAP( college of American Pathologists) guidelines.
